# Airborne exposure-induced occupational type I allergy to Tenebrio molitor: Case report and studies on immunological reactivity 

**DOI:** 10.5414/ALX02580E

**Published:** 2025-07-22

**Authors:** Laura Weißenborn, Sabine Kespohl, Silke Maryska, Ingrid Sander, Jakob Bickhardt, Thomas Henle, Monika Raulf

**Affiliations:** 1 Chair of Food Chemistry, Technische Universität Dresden, Dresden,; 2 Institute for Prevention and Occupational Medicine of the German Social Accident Insurance, Institute of the Ruhr University Bochum (IPA), Bochum, and; 3 Pulmonary Specialist Practice and Training Center, Dresden, Germany

**Keywords:** airborne exposure, occupational allergy, Tenebrio molitor, specific IgE, workplace

## Abstract

This occupational case report describes a 28-year-old woman, employed since November 2020 as a research assistant working with mealworms (*Tenebrio molitor*), who developed symptoms of mild shortness of breath and severe redness and swelling of the eyes in early 2022, leading to the diagnosis of occupational allergic asthma due to respiratory sensitization to mealworm. Subsequent tests confirmed mono-sensitization to *T. molitor* and additional cross-sensitization to flour beetle (*Tribolium confusum*). Further studies aimed to determine the antigenic and allergenic potency of different protein fractions and rearing material isolated from *T. molitor* regarding protein pattern and immunological activity by *T. molitor*-specific polyclonal rabbit IgG and human IgE. The highest antigen content of *T. molitor* was measured in the rearing material, followed by mealworm flour and aqueous extraction. Allergenic proteins are particularly detected in mealworm flour and aqueous fraction at molecular weights of 24 and 11 kDa, which appear to be specific for primary airway sensitization. In addition, the airborne *T. molitor* antigen levels in the workplace were monitored using electrostatic dust collectors to identify hotspots of exposure. The disposal of dry insect material was shown to be responsible for the unintentional release of potential allergens. Therefore, awareness of the potential risk of a type I allergy due to the release of airborne insect allergens in the workplace should be raised.

## Introduction 

Edible insects are considered a sustainable, alternative source of protein. Four insect species are currently approved as food in the EU by the European Food Safety Authority (EFSA) (as of 03/2025): migratory locust (*Locusta migratoria)*, house cricket (*Acheta domesticus),* the larvae of lesser mealworm beetle (*Alphitobius diaperinus)*, also known as buffalo worm, and the larvae of yellow mealworm beetle (*Tenebrio molitor)*. For example, the heat-dried larvae of the mealworm beetle *T. molitor,* the first insect to be approved in 2021 as a food in Europe, can be sold either whole or ground, or processed into biscuits or pastries. 

There is consumer skepticism regarding the acceptance of insects as food; however, several sources indicate that the willingness to consume insects increases when the insects are offered in a processed, non-visible form [1, 2]. Consequently, research is being conducted into the potential use of mealworm protein fractions as a techno-functional food ingredient, analogous to milk proteins, for example to improve the gelling or emulsifying properties of vegetarian substitute products. 

In addition to the potential that insects have as a “protein source of the future”, an allergenic risk must also be considered. In 2021, the EFSA stated for frozen and dried preparations of whole yellow mealworm larvae that consumption may trigger primary IgE-mediated sensitization and subsequent allergic reaction [3]. Allergies can occur particularly among people with pre-existing sensitization to crustaceans or house dust mites. The Panel on Nutrition, Novel Foods and Food Allergens (NDA) concluded its statement with the recommendation to further investigate the allergenicity of the yellow mealworm, including its cross-reactivity to other known allergens. This has recently been confirmed in recital 9 of the Commission Implementing Regulation (EU) 2025/89 [4], in which the authority recommended to carry out further research on the allergenicity of *T. molitor* larvae. 

The EFSA statement only refers to the risk of food allergies for consumers through consumption of insect proteins. In addition, sensitization can also occur through respiratory exposure to insect proteins, for example through occupational exposure. This concerns also workplaces in food and/or feed processing industries, where insects themselves, insect proteins, and the above-mentioned nutrition additives from mealworm can cause a respiratory occupational exposure to insects. For mealworms, 4 clinical case reports of inhalation allergy in insect breeders due to occupational exposure were presented [5]. In addition to respiratory conditions, symptoms of a food allergy to *T. molitor* have been described. In 3 of the 4 affected persons, primary sensitization to yellow mealworm beetle larvae was assumed based on the tests performed. 

The market for insect-based products is expected to grow rapidly to a production of 260,000 tons by 2030 [6]. In addition to the actual breeding farms, increased use in the food industry is also to be expected. Since February 2025, UV-treated mealworm powders may be used in bread and rolls, cakes, pasta, cheese products, and fruit compotes, among other things, which includes a wide range of manufacturing jobs. Apart from this, there are also areas of application as feed and pet food, so that numerous workplaces can be expected to have a potential risk of sensitization to insects. 

Here we present the case of an IgE-mediated sensitization to the mealworm *T. molitor* during occupational exposure in a research lab. The aim of the subsequent investigations was to estimate the allergenic potential of *T. molitor* by analyzing the protein fractions of *T. molitor* and the rearing material. In addition, airborne *T. molitor* exposure was measured at the workplace to detect hotspots of exposure. 

## Case report 

### Occupational history, onset of symptoms, and medical examinations 

Since 11/2020, a 28-year-old woman without atopic history has been employed as a research assistant in the food sector with research focus on mealworms (*T. molitor*). The exposure to mealworms started already in 10/2019 at the beginning of her scientific thesis. In spring 2021, dyspnea occurred for the first time during grinding of the insects. Prior to this, in early 2021, the production process to obtain mealworm material was optimized to produce a much finer mealworm flour. The patient ingested mealworms once in the summer of 2021 without any allergic reaction and no allergic problems known regarding the consumption of crustaceans or the sensitization to mite allergens. 

In 2021, a further episode of shortness of breath occurred. However, in early 2022, mild shortness of breath along with severe redness and swelling of the eyes occurred repeatedly over ~ 6 weeks at the workplace. As a result, the responsible occupational health physician was informed, and initial examinations were conducted. Prior to this, there had been no history of allergies or asthma. To assess IgE-mediated sensitization, a prick test was carried out including allergens from tree and grass pollen, house dust mite, molds, and dog epithelia, all with negative results. However, a prepared solution from mealworm flour (*T. molitor*, provided by the patient) mixed with double-distilled water induced a skin reaction of 16 mm wheal and 40 mm erythema, indicating IgE sensitization. Corresponding serological results using the ImmunoCAP system 250 (ThermoFisher Scientific, Phadia AB, Uppsala, Sweden) supported sensitization to *T. molitor* with CAP class 4. No IgE sensitization was measurable to tree/grass pollen, molds, animal dander, mites, shrimp, and recombinant tropomyosin from *Dermatophagoides pteronyssinus* (Der p 10) and rPen a 1 (shrimp, *Penaeus aztecus*). The total IgE concentration was 154 kU/L. Bodyplethysmographic measurement was normal and the methacholine challenge showed unspecific bronchial hyperresponsiveness. The current examination results raised the suspicion of an occupational disease (classified as number 4301 according to German regulations) attributed to *T. molitor* and were reported to the accident insurance fund. 

Further examinations by a specialized pneumologist later confirmed the previous mono-sensitization to *T. molitor*, now at 5.1 kUA/L (CAP class 3) and additional IgE sensitization to confused flour beetle (*Tribolium confusum*) at 1.74 kUA/L (CAP class 2). Specific IgE to house dust mite and flour moth were still below 0.35 kUA/L, and the total IgE level was 73 kUA/L, all values were carried out with ImmunoCAP system 250. The final diagnosis of occupational allergic asthma, corresponding to occupational disease number 4301, was made. 

### Estimation of the allergenic and antigenic potency of *T. molitor* preparations 

For further characterization of the antigenic and allergenic potency of *T. molitor*, flour, different protein fractions, plus rearing material were investigated. All these samples were part of the regular scientific work in the laboratory carried out by the patient before IgE-mediated sensitization occurred. 

**Protein fractionation procedure**

Mealworm larvae were obtained from a local pet supply store and fasted on sieves for 24 hours. During the fasting period, the excretions and other sieved material such as pieces of chitin shells produced after molting, were collected, which are referred to below as “rearing material”. After killing by blanching, which is a common method in the food industry to reduce the microbial load, the larvae were freeze-dried, defatted using n-hexane (w/v 1 : 5) and then ground into flour. 

**Figure 1.**
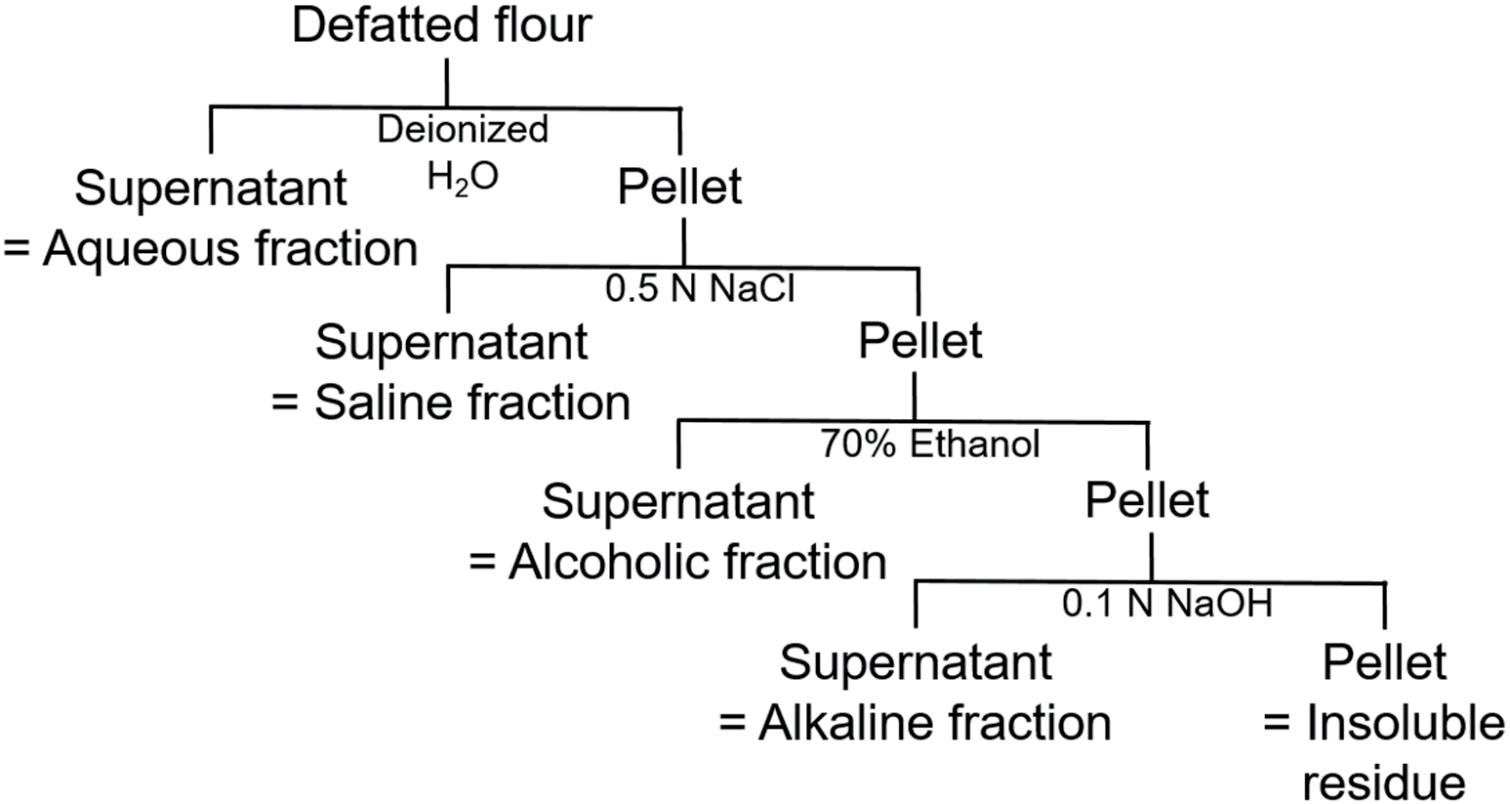
Scheme of the fractionated extraction of mealworm flour according to Osborne (1907) [8].

Protein extraction was performed by stepwise fractionation based on the Osborne fractionation (Figure 1) method for cereal proteins [7] with slight modifications. Thus, four protein fractions and an insoluble residue were obtained, which were subsequently lyophilized. 

**Characterization of protein content and pattern of *T. molitor* fractions**

Protein concentration was measured in all prepared extracts (dissolving 10 mg/mL flour or lyophilizates and rearing samples, excluding the alcoholic fraction of which 25 mg/mL was used due to a low expected protein content) using the Bradford assay (Bio-Rad, Munich, Germany) with bovine serum albumin as reference standard according to the manufacturer’s instructions. Soluble protein contents ranged from 2 to 41% (Table 1). 

**Figure 2.**
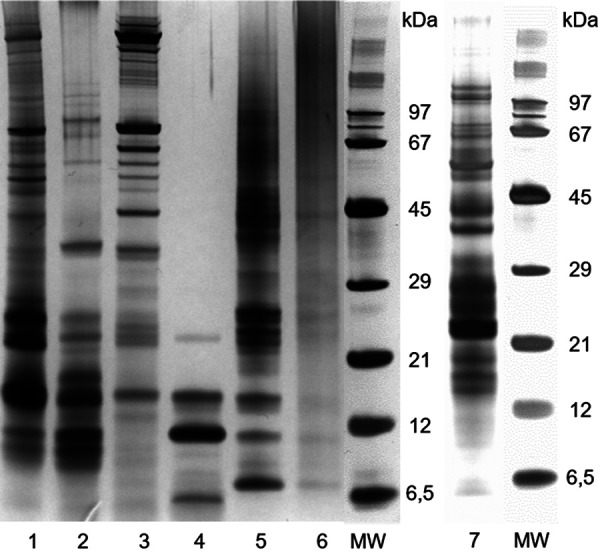
Silver-stained protein patterns after SDS-PAGE of *Tenebrio molitor* samples. Each lane was loaded with 1.5 μg of protein. 1 = defatted flour; 2 = aqueous fraction; 3 = saline fraction; 4 = alcoholic fraction; 5 = alkaline fraction; 6 = insoluble residue; 7 = rearing material; MW = molecular weight of marker proteins.

The protein pattern of mealworm samples was analyzed using sodium dodecyl sulphate-polyacrylamide gel electrophoresis (SDS-PAGE) according to Kespohl et al. [8] with a final protein content of 1.5 µg/lane. Non-quantitative silver staining was performed with minor modifications according to Pierce Silver Stain Kit (Thermo Scientific (Figure 2). 

Water-soluble protein fractions including defatted mealworm flour extract, aqueous fraction and saline fraction showed comparable protein pattern with numerous bands in the range of 10 – 70 kDa (Figure 2, lanes 1 – 3). The alcoholic fraction showed only proteins below 21 kDa, with one characteristic band ~ 10 kDa (Figure 2, lane 4). The alkaline fraction exhibited numerous protein bands over the entire molecular size range, probably due to cross-linking reactions during extraction under alkaline conditions (Figure 2, lane 5). The insoluble residue had no clear protein bands but was smeared with residual bands from the alkaline fraction. The rearing material (Figure 2, lane 7) showed numerous bands between 12 and 100 kDa. Particularly evident is a band ~ 25 kDa in the rearing material, which was similar to the bands in the blanched flour. 

**Analysis of the antigen and allergen content in *T. molitor* fractions**

All samples were measured by a specific sandwich ELISA using anti-*T. molitor* polyclonal antibodies to determine the specific *T. molitor* antigen content in each fraction. Briefly, *T. molitor* larvae purchased from a pet shop were starved, washed, crushed, centrifuged, and the supernatant was used for immunization to raise rabbit antibodies. Antibodies were affinity purified from rabbit serum for sandwich immunoassay development. For the standard curve, aliquots of the larval preparation were diluted to 1 µg/mL and stabilized with 1% casein. The measuring range of the immunoassay was 0.3 –  100 ng/mL based on the protein content of the standard preparation (Sander et al., manuscript in preparation). 

**Figure 3.**
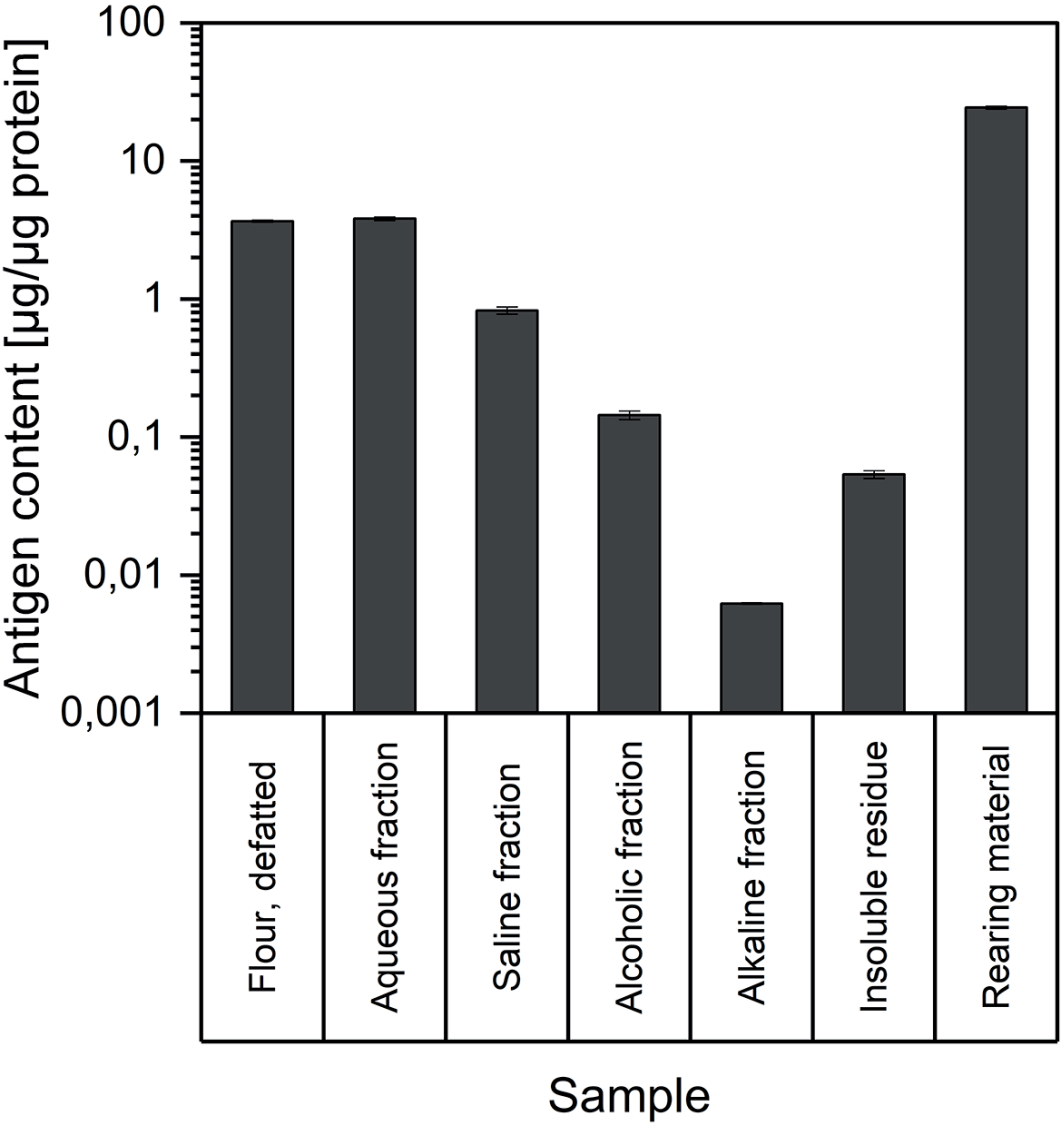
Antigen levels of* Tenebrio molitor *samples, quantified by* Tenebrio molitor-*specific sandwich ELISA.

The flour sample and the aqueous fraction showed high antigen levels between 3.7 and 3.8 µg/µg protein (Figure 3). Antigen levels of around 0.8 µg/µg protein were found in the salty fraction. In the remaining fractionation samples, the *T. molitor* antigen content was below 0.2 µg/µg protein, with the lowest values of 0.006 µg/µg protein in the alkaline Osborne fraction of the blanched samples. The quantification of antigens in the samples showed that the allergenic potential decreases during the fractionated extraction, as carried out in the patient’s laboratory. This is consistent with the assumption that antigens and allergens are generally highly soluble in water and have therefore been extracted in the first fractions. An alkaline extract could possibly be considered as a fraction with lower allergen content. The highest content of 24.4 µg/µg protein was determined for the rearing material (excrements, chitin shells as products of molting, etc.) (Figure 3). These are valuable findings regarding occupational health and safety. Particularly relevant for insect farms is the fact that the rearing material is produced in large quantities during rearing, as the processes of fasting or keeping on sieves are used as standard practice to separate the excretions from the insects. 

**Immunoblotting**

For the detection of IgE-binding proteins, the PVDF blots were incubated with the serum of the patient sensitized to *T. molitor* as primary antibody, and with *T. molitor*-specific polyclonal antibodies (Sander et al., manuscript in preparation) for IgG-binding antigens as described elsewhere [8]. 

**Figure 4.**
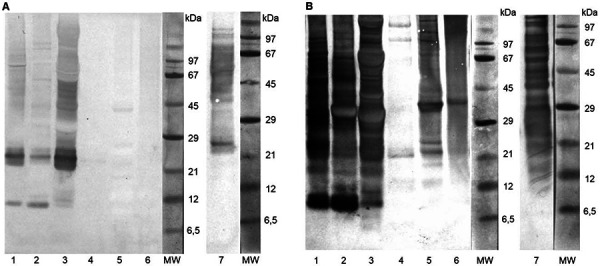
Human IgE antigen profile (A) and rabbit IgG antigen profile (B) of immunoblots of* Tenebrio molitor *samples. 1 = defatted flour; 2 = aqueous fraction; 3 = saline fraction; 4 = alcoholic fraction; 5 = alkaline fraction; 6 = insoluble residue; 7 = rearing material; MW = molecular weight of marker proteins.

In the human IgE blot, IgE-binding structures could only be identified in flour, aqueous and salty fractions of blanched mealworms (Figure 4A, lanes 1 – 3), and in the rearing material (Figure 4A, lane 7). The most prominent bands were at 24 kDa as well as at 11 and 53 kDa. No protein bands were identified in the other protein fractions and the residue with the serum of the sensitized person. 

Antigenic structures identified by incubation of the blots with *T. molitor*-specific rabbit IgG were more numerous and present over a wide molecular weight range. Only the alcoholic fraction showed hardly any bands. The most intense patterns were found in the samples also detected in the human IgE blot (Figure 4A, B, lanes 1 – 3, lane 7), with corresponding bands of the allergenic structures at 11 and 24 kDa. 

Regarding the identified allergens for *T. molitor*, there is currently no entry in the allergen database of WHO/IUIS Allergen Nomenclature Subcommittee (www.allergen.org), neither as a food allergen nor as an inhalation allergen [9]. In the case of existing sensitization to crustaceans or house dust mites, it is suspected that cross-reactive pan-allergens such as tropomyosin or arginine kinase might be responsible for IgE sensitization to *T. molitor*. In a study by Broekman et al. [10], human IgE blots showed that tropomyosin and arginine kinase homologues were the most relevant allergens for patients with an existing shrimp allergy. In patients with primary sensitization to mealworm, other proteins such as larval cuticle proteins or chitin-binding protein were responsible for allergenicity [5]. Another study by Nebbia et al. [11] postulated that cockroach allergen-like protein is involved in primary respiratory and food allergy to *T. molitor*. As it is considered to be an isoallergen by cleavage by trypsin-like enzymes present in the cockroach gut, the reported molecular weight varies from 6 to 37 kDa [12]. The molecular weights for known potential cross-allergens are 32.8 kDa for tropomyosin, 40 kDa for arginine kinase, 26.5 kDa for cationic trypsin, and 50.6 and 51.7 kDa for tubulin α-1 chain and α-amylase [13]. Only the cationic trypsin was in the same molecular weight range as the 24-kDa IgE-binding protein, but this would need to be confirmed by mass spectrometry and has not yet been described as a potential mealworm allergen for primary respiratory sensitization. Ganseman et al. [14] also showed IgE binding of an insect-exposed worker to a 25-kDa mealworm allergen, which was not further characterized. The molecular weight of the reported *T. molitor* IgE-binding proteins mentioned above corresponds to the findings of the present case report. Here, the patient was sensitized by respiratory exposure during the processing of mealworm larvae, and IgE-binding from the patient’s serum showed dominant allergens with molecular weights of 11 and 24 kDa in the IgE blot. Especially in case of respiratory IgE-mediated sensitization by insects, further research approaches are important. Therefore, further studies on the allergenicity of larvae from *T. molitor* and insects processed in Germany and the European Union should be carried out, as recommended by EFSA [3]. 

### Airborne *T. molitor* monitoring at the workplace to detect the hot spots of exposure 

**Figure 5.**
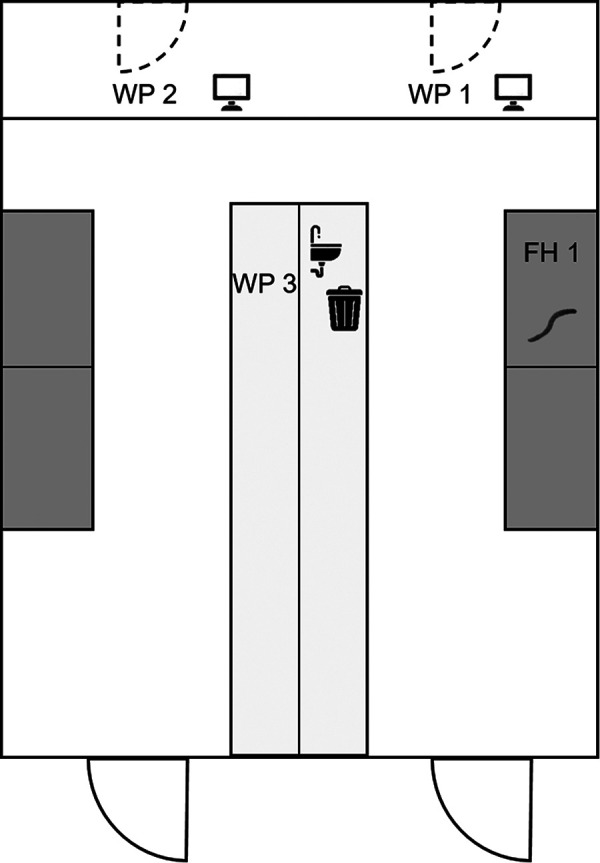
Floor plan of the laboratory for the airborne dust collection. Dark gray: fume hoods, light gray: workbenches/documentation desks. WP 1 – 3: Workplaces 1 – 3, where the passive samplers were laid out. FH 1: Fume hood, in which the work with the living and dry mealworm samples usually took place.

For monitoring of airborne antigen levels at the workplace of the *T. molitor*-sensitized patient, passive dust sampling was performed with so-called “electrostatic dust collectors” (EDCs) [15, 16] during typical laboratory work with mealworms (Figure 5). Workplaces 1 and 2 were located at the windows on the left and right of the laboratory, above documentation desks. The EDC at workplace 3 was placed on a shelf in the left half of the laboratory. The dust was collected for 2 weeks in 2 periods, before and after an intervention to reduce mealworm exposure, with comparable activities being carried out in the laboratory. 

The extraction of the EDC cloths was carried out according to Zahradnik et al. [16]. In brief, one EDC cloth was extracted with 15 mL extraction buffer (PBST, 0.05% Tween) for 1 hour, then centrifuged and the supernatant stored at –80 °C until quantification by sandwich ELISA (Sander et al., manuscript in preparation). 

**Figure 6.**
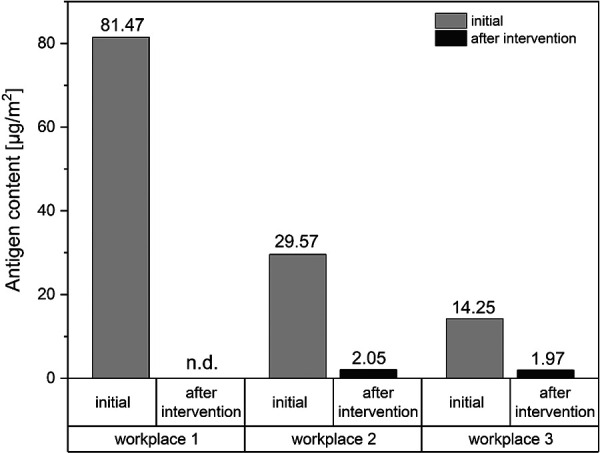
Antigen contents of the workplace exposure measurement (see Figure 5) before and after intervention, measured by electrostatic dust collectors, quantified by *Tenebrio molitor*-specific sandwich ELISA.

During collection of *T. molitor* antigens, living insects were processed, fasted for 24 hours, and sorted. All work steps with the mealworms were carried out in the fume hood (FH 1) (Figure 5) as long as they were dry. The critical steps of grinding and filling the freeze-dried extracts also took place in FH 1. Nevertheless, high antigen levels ranging from 14.25 to 81.47 µg/m^2^ were measured at all locations in the laboratory during the initial measurement. The results of the passive air sampling are shown in Figure 6. It is assumed that the maximum *T. molitor* antigen content/m^2^ is due to the proximity of the EDC at workplace 1 to the laboratory waste (Figure 5), where the sorted material (mainly chitin shells and excretions) was disposed. This is consistent with the results of antigen quantification, in which the highest levels were determined in the rearing material. Therefore, as an intervention to reduce antigen exposure to airborne dust during laboratory work, dry, sorted material, as well as any kind of waste, was collected in the fume hood and soaked before disposal in the laboratory waste. In addition, all equipment (spatulas, glass containers, etc.) was rinsed in the fume hood after use, eliminating the risk of potentially allergenic dust being released. The success of this intervention is evident in the post-intervention data, where the maximum exposure is 2.05 µg/ m^2^, although comparable work was carried out for the initial measurement. It was also found that there was no carryover of airborne antigens into the offices at any time. 

The measurements of the airborne dust in the workplace showed that a risk assessment of the workplace is appropriate in terms of occupational health and safety. By monitoring antigen exposure in individual settings, uncontrolled exposure to potential allergens can be avoided. In the current case, decontamination of the hazardous material in the fume hood was sufficient. Occupational safety measures should be applied according to the “STOP” principle: Substitution, Technical Protective Action, Organisational Protective Action, and Personal Protective Action [17]. In addition to the technical protective action at the workplace, personal protective equipment such as FFP3 masks may be recommended. 

These findings are consistent with those of Ganseman et al. [14] who studied 15 insect-exposed workers and reported on the incidence of occupational allergies and the prevalence of sensitization to insects. They found that 60% of the workers reported upper respiratory symptoms related to insect exposure. Ten workers (71.4%) had a positive histamine provocation test concentration of less than 8 mg/mL, resulting in a 20% decrease in FEV_1_. In addition to stationary measurements, personal air sampling was taken during their air dust measurement. By installing ventilation at the sifting station (separating the mealworms from their waste) and introducing personal protective equipment (disposable lab coats and full-face masks with P3 dust filters), they were able to significantly reduce exposure to airborne dust, confirming the need for infrastructural adjustments to occupational health and safety. They recommend the introduction of limit values for potentially allergenic dusts, especially for this industry. 

## Summary and perspective 

This case report demonstrates primary sensitization to *T. molitor* in a non-atopic laboratory research scientist diagnosed with occupational allergic asthma under occupational disease number 4301. The example of the mealworm shows that insects must be considered as new sources of occupational respiratory allergens. High antigenic and allergenic potential was found for mealworm flour, protein fractions, and rearing material. Especially the rearing material showed a high antigen content, bearing a potential risk for occupational exposure and sensitization in insect farms. 

Two proteins of 11 and 24 kDa were identified as the most important IgE-binding structures in the current case. Further studies to identify allergens in *T. molitor* are necessary for a reliable diagnosis of occupational allergy and subsequent tailor-made prevention strategies at the workplaces, as well as to clarify potential cross-reactions to food allergens. For consumers, cross-reactivity of certain allergens may be particularly important in the case of pre-existing sensitization to crustaceans and/or house dust mites, whereas occupational exposure may lead to new sensitization without the involvement of potential cross-allergens. 

Regarding the expected increase in production and the expanding field of insect processing, risk assessment of workplaces seems important and awareness of the occurrence of respiratory symptoms caused by working with insects should be increased in the occupational health sector. 

## Authors’ contributions 

Conception and design of the study: MR, SK, LW, TH. 

Data collection: SM, LW, SK, JB. 

Data analysis and interpretation: LW, SK, SM, IS, JB, TH, MR. 

Manuscript drafting: LW, SK, SM, IS, JB, TH, MR. 

## Funding 

This research did not receive any specific grant from funding agencies in the public, commercial, or not-for-profit sectors. The data were collected as part of investigation within the scope of claims for compensation due to occupational asthma at the Institute for Prevention and Occupational Medicine, Institute of the Ruhr University Bochum (IPA) which is financed by the German Social Accident Insurance (DGUV). 

## Conflict of interest 

All authors declare that there is no conflict of interest with regard to this work. 

**Table 1.** Total protein content of aqueous extracts of Tenebrio molitor samples and soluble protein content.

**Sample**	**Protein concentration of extracts [µg/mL]**	**Soluble protein content in lyophilized fraction [%]**
Flour, defatted	614	6.1
Aqueous fraction	1,022	10.2
Saline fraction	4,095	40.6
Alcoholic fraction*	504	2.0
Alkaline fraction	775	7.8
Insoluble residue	1,770	17.7
Rearing material	290	2.9

*Use of 2.5 times the amount of lyophilizate due to a low expected protein content.

## References

[1] Balzan S Fasolato L Maniero S Novelli E Edible insects and young adults in a north-east Italian city - an exploratory study. Br Food J. 2016; 118: 318–326.

[2] Bußler S Rumpold BA Jander E Rawel HM Schlüter OK Recovery and techno-functionality of flours and proteins from two edible insect species: Meal worm (Tenebrio molitor) and black soldier fly (Hermetia illucens) larvae. Heliyon. 2016; 2: e00218. 28054035 10.1016/j.heliyon.2016.e00218 PMC5198854

[3] Turck D Bohn T Castenmiller J De Henauw S Hirsch-Ernst KI Maciuk A Mangelsdorf I McArdle HJ Naska A Pelaez C Pentieva K Siani A Thies F Tsabouri S Vinceti M Cubadda F Frenzel T Heinonen M Marchelli R Neuhäuser-Berthold M Safety of frozen and dried formulations from whole yellow mealworm (Tenebrio molitor larva) as a novel food pursuant to Regulation (EU) 2015/2283. EFSA J. 2021; 19: e06778. 34466159 10.2903/j.efsa.2021.6778 PMC8385682

[4] Commission Implementing Regulation (EU) 2025/89 of January 20, 2025 authorising the placing on the market of UV-treated powder of whole Tenebrio molitor larvae (yellow mealworm) as a novel food and amending Implementing Regulation (EU) 2017/2470.

[5] Broekman HCHP Knulst AC den Hartog Jager CF van Bilsen JHM Raymakers FML Kruizinga AG Gaspari M Gabriele C Bruijnzeel-Koomen CAFM Houben GF Verhoeckx KCM Primary respiratory and food allergy to mealworm. J Allergy Clin Immunol. 2017; 140: 600–603. 28274747 10.1016/j.jaci.2017.01.035

[6] International Platform of Insects for Food and Feed (IPIFF). Edible insects on the European market. 2020; https://ipiff.org/wp-content/upl​o​a​d​s​​/​2​0​20/06/10-06-2020-IPIFF-edible-insects-mark​e​t​-​f​actsheet.pdf [04/03/2025].

[7] Osborne TB The Proteins of the Wheat Kernel. Carnegie Institute, Washington D.C., 1907.

[8] Kespohl S Maryska S Zahradnik E Sander I Brüning T Raulf-Heimsoth M Biochemical and immunological analysis of mould skin prick test solution: current status of standardization. Clin Exp Allergy. 2013; 43: 1286–1296. 24152161 10.1111/cea.12186

[9] ALLERGEN NOMENCLATURE, WHO/IUIS Allergen Nomenclature Sub-Committee, allergen.org [March 4, 2025].

[10] Broekman HCHP Knulst AC de Jong G Gaspari M den Hartog Jager CF Houben GF Verhoeckx KCM Is mealworm or shrimp allergy indicative for food allergy to insects? Mol Nutr Food Res. 2017; 61: 1601061. 10.1002/mnfr.201601061 28500661

[11] Nebbia S Lamberti C Giorgis V Giuffrida MG Manfredi M Marengo E Pessione E Schiavone A Boita M Brussino L Cavallarin L Rolla G The cockroach allergen-like protein is involved in primary respiratory and food allergy to yellow mealworm (Tenebrio molitor). Clin Exp Allergy. 2019; 49: 1379–1382. 31309657 10.1111/cea.13461

[12] Mueller GA Pedersen LC Lih FB Glesner J Moon AF Chapman MD Tomer KB London RE Pomés A The novel structure of the cockroach allergen Bla g 1 has implications for allergenicity and exposure assessment. J Allergy Clin Immunol. 2013; 132: 1420–1426. 23915714 10.1016/j.jaci.2013.06.014 PMC3844097

[13] Verhoeckx KCM van Broekhoven S den Hartog-Jager CF Gaspari M de Jong GAH Wichers HJ van Hoffen E Houben GF Knulst AC House dust mite (Der p 10) and crustacean allergic patients may react to food containing Yellow mealworm proteins. Food Chem Toxicol. 2014; 65: 364–373. 24412559 10.1016/j.fct.2013.12.049

[14] Ganseman E Goossens J Blanter M Jonckheere AC Bergmans N Vanbrabant L Gouwy M Ronsmans S Vandenbroeck S Dupont LJ Vanoirbeek J Bullens DMA Breynaert C Proost P Schrijvers R Frequent Allergic Sensitization to Farmed Edible Insects in Exposed Employees. J Allergy Clin Immunol Pract. 2023; 11: 3732–3741.e10. 37543086 10.1016/j.jaip.2023.07.039

[15] Krop EJ Jacobs JH Sander I Raulf-Heimsoth M Heederik DJ Allergens and β-glucans in dutch homes and schools: characterizing airborne levels. PLoS One. 2014; 9: e88871. 24551183 10.1371/journal.pone.0088871 PMC3925184

[16] Zahradnik E Sander I Kendzia B Fleischer C Brüning T Raulf-Heimsoth M Passive airborne dust sampling to assess mite antigen exposure in farming environments. J Environ Monit. 2011; 13: 2638–2644. 21842065 10.1039/c1em10430f

[17] BG Verkehr. STOP-Prinzip. https://www.bg-verkehr.de/arbeitssicherheit-gesundheit/themen/gefahrstoffe/schutzmassnahmen/stop-prinzip [March 4, 2025].

